# Using population registers for migration and integration research: examples from Denmark and Sweden

**DOI:** 10.1186/s40878-018-0076-4

**Published:** 2018-06-18

**Authors:** Romana Careja, Pieter Bevelander

**Affiliations:** 1 University of Southern Denmark, Campusvej 55, 5230 Odense, Denmark; 2 Malmö Institute of Migration, Diversity and Welfare, 71 Malmö högskola, Kultur och samhälle, 205 06 Malmö, Sweden

**Keywords:** Register-based information, Register-based sampling, Immigrants, Integration, Sweden, Denmark

## Abstract

The paper starts from the observation that research on immigrants’ integration trajectories needs detailed information, both objective and attitudinal, and ideally longitudinal. This study uses the cases of Denmark and Sweden – whose registers produce detailed records about all natives’ and immigrants’ lives in their host countries – in order to, first, review existing research on immigrants and their integration and, second, discuss the way in which register data are used, their caveats and their potential. The study finds that, in Denmark and Sweden, registers provide systematic objective data which are fully available to researchers and have the potential to help in the collection of high-quality subjective data. However, the population registers have some traits which may impact on the representativeness of the samples. The authors argue that, if researchers are aware of the caveats, registers can be used to obtain representative samples of immigrants, and register data can be complemented with survey-based attitudinal data, thus opening up new research opportunities for testing propositions on integration theories.

## Introduction

From social cohesion to diversity, and from inequality to identity formation, immigrants’ integration challenges many contemporary European societies. Understanding how integration unfolds, what influences it and how it, in turn, influences other phenomena requires not only a great deal of new data but also a very specific type of data. It is not the aim of this paper to produce a comprehensive evaluation of all the available immigrant data. High-level discussions between academics, policy-makers and other stake-holders, such as those taking place currently under the aegis of the International Forum of Migration Statistics 2018, highlight that we are currently witnessing a very dynamic process which goes beyond simply immigrant data collection and opens up new spaces for reflection and action related to new methods of data collection, new data sources and questions of ethics and the use of data. Based on such debates and on existing research it is possible to make several observations about the state of data availability, in particular data that enable scholars to conduct research focused on integration:

1. *Availability* to researchers: it is clear that more and more data about and from immigrants are collected by governments, international organizations and research institutes, as the discussions at the International Forum on Migration Statistics 2018 have evidenced. However, not all these data are available to researchers. A great deal of data collected by governments are protected under various legal frameworks, while data collected by research groups are not made public for certain periods of time. Most individual-level data on immigrants which are currently publicly available come from large survey programs like ESS, LFS, or EVS/WVS, or country-specific surveys.
2. *Systematic* data collection: owing very much to financial constraints as well as other practical considerations (for reflections on the difficulties of collecting immigrant data, see Fassmann, Reeger, & Sievers, 2009; Font & Mendez, 2013; Groenewold & Lessard-Phillips, 2012), data collection on immigrants differs dramatically across countries (as the contributions to this special issue have also illustrated; see also Fassmann et al., 2009). At one end of the spectrum, Scandinavian countries systematically collect a great deal of administrative data on the entire immigrant population (for details, see below). At the other end of the spectrum are countries which do not have population registers. In such situations, scholars obtain immigrant data from surveys. However, only a few countries implement programs which systematically survey immigrant populations. For example, only Germany, the Netherlands or the UK have large-scale survey programs which collect data at regular time intervals *and* which implement procedures to extract representative immigrant samples. Most countries implement less-systematic immigrant data collection and rely on data produced by general population surveys or by immigrant surveys conducted every now and then. As to the time dimension, most immigrant survey data are crossectional, with only a precious few countries implementing immigrant panels. Although theoretically some immigrant panel data can be obtained from panel surveys of the general population, in practice it is problematic, as immigrants have a much higher drop-out rate than natives (Dustmann & Weiss, 2007; Edin, LaLonde, & Åslund, 2000; Warrent & Peck, 1980).
3. *Representativeness* of immigrant samples: despite their public availability, a good many survey data on immigrants have a significant flaw. As has become clear from the observations above, with few significant exceptions, most of these data come from surveys based on representative samples of the general population. In addition to including comparatively few immigrants (and their children), these general population samples do not produce *representative* samples of the immigrant population in the respective countries.
4. *Objective*^1^ and *subjective* data: a bird’s-eye view of integration research in Europe reveals a dominance of studies focusing on the socio-economic characteristics and achievements of immigrants. Numerous studies^2^ compare immigrant groups to natives (Algan, Dustmann, Glitz, & Manning, 2010; Crul & Doomernik, 2003; Dahlstedt & Bevelander, 2010; Husted, Nielsen, Rosholm, & Smith, 2001; Kogan, 2006; OECD, 2015; Pichler, 2011) or to each other (Crul, 2013; Crul & Doomernik, 2003; Crul & Vermeulen, 2003; Crul, Schneider, & Lelie, 2012; Dribe & Lundh, 2008; Fleischmann, Phalet, & Klein, 2011; Güveli, 2015; Phalet & Schönpflug, 2001; Silberman, Alba, & Fournier, 2007; Vermeulen, 2010), while others emphasize the role of contextual factors for immigrants’ life chances (Crul & Schneider, 2010; Crul et al., 2012; Van Tubergen, Maas, & Flap, 2004; for a slightly different take, see Ersanili & Koopmans, 2011). In contrast, fewer studies focus on attitudes and preferences of immigrants themselves as indicators of their embeddedness in host societies (or absence thereof). Without claims of exhaustivity, we note contributions focusing on immigrants’ preferences for redistribution (Luttmer & Singhal, 2011; Schmidt-Catran & Careja, 2017), on (dis)identification with the host nation and trust in institutions (De Vroome, Coenders, van Tubergen, & Verkuyten, 2011; De Vroome, Martinovic, & Verkuyten, 2014; Dinesen & Hooghe, 2010; Fokkema & de Haas, 2015; Röder & Mühlau, 2011, 2012, 2014; Verkuyten & Martinovic, 2012), on political and civic engagement (Aleksynska, 2011; de Rooij, 2012; Morales & Giugni, 2011) or on attitudes towards immigrants (Just & Anderson, 2014; Van der Zwan, Bles, & Lubbers, 2017).

Arguably, the dominance of studies based on objective data is due not only to data availability but also to the specificities of subjective data. On the one hand, more data about employment status, income and education levels of immigrants exist compared to attitudinal data. For example, statistical information collected by government agencies become huge repositories of employment status and income information but not of individuals’ attitudes. Even longitudinal surveys with immigrant samples, such as the German socio-economic panel (which oversamples immigrants from the 1980s on), the Dutch LISS immigrant panel (see also Salentin & Schmeets, 2017) or the UK Household Longitudinal Survey,^3^ collect data on large batteries of economic indicators on a yearly basis, while attitudinal questions are not collected every year. On the other hand, attitudinal data are more sensitive to various forms of bias which are not present in objective socio-economic data, which may prompt researchers to use them less: for example, in the existing surveys the attitudinal questions may reflect the perspective and the preferences of the initiators of the survey, and may not correspond to the interests of other researchers.

This brief overview suggests that, when it comes to the immigrant data available to scholars, they are not systematic (with a few notable exceptions), they contain limited information on immigrants’ attitudes and preferences and are rarely based on representative samples of immigrants. The main reason why representative samples of immigrants are difficult (and therefore costly) to obtain is that comprehensive sampling frames are difficult to come by.

The question which emerges is whether having access to data (re)sources which cover the entire population provides a remedy for the data problems mentioned above. In order to provide an answer, this article examines the cases of Denmark and Sweden. The two countries have built a complex system of domain-specific registers (databases of records of *all legally residing* individuals) connected to a central population register (for details, see Danish and Swedish population registers as sources of research data: possibilities and caveats section). Under certain conditions, these registers are available to researchers. Having access to the entire population in the registers provides researchers with rich *objective* data on immigrants and with an ideal sampling frame from which to extract high-quality immigrant samples for surveys (as sources of *subjective* data). In other words, Denmark and Sweden are the most likely countries for obtaining accurate and detailed data on immigrants and, therefore, for observing whether access to these data remedies many of the challenges faced by scholarly research on integration.

The paper builds on information collected from three main sources: documentation from statistical and governmental bodies, a systematic literature research and expert interviews with specialists on register data and survey experts in the two countries. It is undeniable that the availability of register data to scholars outside governmental institutions puts the research context in these two countries in a category of their own. Consequently, the likelihood that the type of research conducted in these two countries can be replicated in other countries is low (notable exceptions are the other Nordic countries, which also have centralized register systems). However, other European countries *do* have population registers which are, to some limited extent, available to researchers (see for example, Salentin & Schmeets, 2017; Sanguilinda, Barbiano di Belgiojoso, Ferrer, Rimoldi, & Blangiardo, 2017). Our analysis will, therefore, speak to the research communities in these countries and to the competent authorities and provide arguments which will hopefully show the benefits of opening up the data in these registers to scholarly research.

The remainder of the article is structured as follows. The paper starts with a brief introduction to the registers in the two countries and a discussion of the characteristics of the data included in them, with particular attention to data concerning immigrants. We then elaborate on how these data are used. Firstly, we present their official use, focusing on the definitions and categories used by the statistical agencies in the two countries. Secondly, we examine how these data are used to research immigrants’ integration. We conclude that, with few exceptions, integration studies are mainly register-based; we then reflect on the advantages and disadvantages of this approach. Thirdly, we discuss the opportunities and caveats of using the registers as sampling frames for immigrant surveys.

## Danish and Swedish population registers

### Denmark

The Danish Civil Registration System (hereafter CRS) is a centralized nation-wide civil register which includes basic personal data for every individual who has received a personal identification number (CPR number). The CRS contains information on all persons residing in Denmark (since 1968) and in Greenland (since 1972). The CRS is updated daily and its maintainance is the responsibility of the Ministry of the Interior, together with the municipalities (Bøcker Pedersen, Gøtzsche, Møller, & Mortensen, 2006).

Data on immigrants are input by the municipalities where they reside. Other authorities, such as the Ministry of Refugee, Immigration and Integration Affairs, the Refugee Appeal Board, the Danish Immigration Service or the Commissioner of the Police and the State Counties, which process residence permits for different categories of aliens, input information into a different register – the Danish Aliens’ Register (*Udlaendingeregistret*). In this register, each person has a specific record number and, if she/he qualifies, also a CPR number through which ensures inclusion in the CRS. This latter depends on residential rights, which depend, in turn, on the country of origin and the reason for an individual’s entry/stay in Denmark. A person can request inclusion in the CRS (and thereby attribution of a CPR number) only if she/he intends to stay longer than three months (in the case of citizens of the European Economic Area – the EEA, in other words EU and EFTA citizens), or more than six months (in the case of citizens of other Nordic countries).^4^

The CRS contains personal information (Bøcker Pedersen et al., 2006; Schmidt, Pedersen, & Sørensen, 2014), and more detailed information can be obtained by linking the CRS with specific registers, such as labour market or health registers. This can be done via the CPR number. Access to these registers is restricted under Data Protection Regulations. Researchers interested in doing register-based research must comply with the regulations and apply to the Data Protection Agency for permission to access the registers.^5^ As a rule, only researchers affiliated with Danish research institutions have access to the data. Foreign researchers can gain access indirectly, through affiliation with a Danish authorised research institution (Statistics Denmark, 2016). For non-affiliated researchers, it is advisable to contact the institutions managing the datasets of interest to find out about conditions of access.^6^

The CRS can be used to identify immigrants because it contains information on *place of birth* and *citizenship*. For all persons living in Denmark or Greenland, the CRS contains information on the full address (municipality, road and house number) and the date when they moved to that address. For immigrants, information on *country of origin* and, for emigrants, information on the country of emigration, is recorded, along with the dates at which this occurred. According to Danish legislation, each resident is obliged to inform the CRS about changes to his or her permanent address within five days of such change occurring. There is a strong incentive to do so, especially for in-country movements, as a failure to communicate this information may result in difficulties (and the outright inability) to access a variety of services and welfare benefits (Bøcker Pedersen et al., 2006). Therefore, CRS data are likely to be accurate (however, see "Danish and Swedish population registers as sources of research data: possibilities and caveats" section for more details). It is also estimated that data collected in other registers (for example, labour market status, income and transfers or health) are equally complete and reliable (Baadsgaard & Quitzau, 2011; Petersson, Baadsgaard, & Thygesen, 2011; Sahl Andersen, de Fine Olivarius, & Krasnik, 2011). The CRS also contains information on *citizenship*. However, register-based citizenship has limited reliability in the identification of immigrants because, when a person receives Danish citizenship, the CRS records only the Danish citizenship and drops the original one. Therefore, naturalization cases can be identified only by looking at long-term data. The register includes only the current citizenship; multiple citizenships are not recorded.

### Sweden

The core of the official statistics system is the Swedish National Population Registration System, administered by the tax authorities. When a person is registered, she or he is given a personal identification number. This number is used for registration in all areas (employment, health and welfare). Both administrative and statistical registers based on individuals, as well as sample surveys, have a personal identification number variable and this facilitates the linkage between the different datasets. Data on individuals are protected under the Secrecy Act. The main principle is that microdata can be accessed in a coded and unidentifiable manner for research purposes if the owner of the data approves the request.

If a researcher wants to use data from a register managed by an authority and wants to link this information to data from Statistics Sweden, the request must be approved by the authority in question as well as by a regional ethical board. If the request is approved, the authority sends the data to Statistics Sweden, with the personal identification number replaced with a sequence number. The authority sends the key to the code to Statistics Sweden, which uses the same code on the data requested from them before sending the coded data to the researcher. The key to the code will be saved for three months at Statistics Sweden. In the Official Statistics Act (2001:99) the possibility to save the key was extended in cases where there might be a special need to complement the material later, during the course of the research project (Statistics Sweden, 2012). As a rule, researchers must be affiliated with a Swedish research institute/university. Foreign researchers may have access if they are affiliated with a Swedish research institution. For non-affiliated researchers, it is advisable to contact the data manager to find out about conditions of access.^7^

As in Denmark, the register includes information that allows the identification of immigrants, primarily by *country of origin/birth* and *citizenship*. As family members can be traced, the second and third generations of immigrants can be identified by looking up the country of origin of the parent(s) and grandparent(s) respectively. As in Denmark, *citizenship* is available, but its usefulness in properly identifying immigrants is limited because, if a person acquires Swedish citizenship, only the Swedish citizenship is recorded, regardless of other citizenships. If a person holds multiple citizenships, only one is recorded.

## Danish and Swedish population registers as sources of research data: possibilities and caveats

As a research tool, Danish and Swedish registers have several undoubted qualities. This section presents these qualities in general, and reflects upon the usefulness of registers as data sources for research on immigrants.

Firstly, the registers provide *complete* information. They provide the researchers with access to the entire population (of the respective administrative unit) which, in the case of Denmark and Sweden, means the entire legally residing population, as the registers are centralized at the national level. The same information is collected on virtually all individuals.

Secondly, the registers provide *longitudinal data*. Information, including precise records of occurrence and duration of events, is crucial for understanding not only individuals’ trajectories but also processes on a more aggregate level. Moreover, by using longitudinal data, it is possible to introduce time-related events/covariates in the analysis and estimate causality between events and behavior.

Thirdly, registers provide *accurate* data. By recording information in predetermined categories and by recording the exact dates of changes, the data included in registers are particularly accurate. To the extent that the administrative definitions remain the same, the data are largely comparable over time. Overall, the data quality is ensured by the quality control protocols in place (Eurostat, 1995; UN, 2007). Moreover, as government agencies routinely use these data, it is likely that errors will be noticed and corrected (Schmidt et al., 2014).

Fourthly, and related to the previous point, registers provide data which are less *sensitive to bias* related to self-reporting (from concerns for privacy to recall problems) compared to surveys. This advantage becomes clearer especially when the topics are considered sensitive or when respondents, for a variety of reasons, feel uncomfortable in an interview situation.

Finally, register data allow the researchers to *avoid bias* associated with sample selection and with non-response (Berk, 1983; Reigneveld & Stronks, 1999) which often affect survey research. As will be shown in "The use of registers: data for immigration research" section, researchers who use immigrant register data often use the entire dataset available, i.e., the entire population of interest.

In addition to these features, which are characteristic of all registers, the *linkability* through the personal identity numbers makes Danish and Swedish registers particularly attractive for researchers – as previously discussed, this feature allows the researcher to pull together (longitudinal) data from registers as diverse as labour market enrolment, education, income, social transfers or health.

There are several reasons why registers can be especially useful for studies focusing on immigrants. Firstly, in strictly practical terms, collecting data on immigrants through surveys can be costly. Moreover, it is fair to say that, although the situation has improved compared to only a decade ago, survey data on immigrants are scarce, as there are relatively few datasets *publicly* available. Some immigrant data are available from large cross-national survey initiatives, like the ESS or national surveys but, unless immigrants are purposefully sampled, their numbers in the final samples are likely to remain small. Although, more recently, several research projects have surveyed immigrants, only a few have made their raw data publicly available. Only a few countries systematically collect data on their immigrant communities. Thus, the readily available register data can save researchers time and money, and provide information about the entire legally residing immigrant population, which strengthens researchers’ ability to make (causal) inferences (Jakobsen & Larsen, 2010).

Secondly, most of the currently available data on immigrants are cross-sectional. Cross-sectional survey data – albeit rich – bring with them specific problems that restrict researchers’ ability to test more complex theoretical arguments. The main weaknesses of cross-sectional studies are their limited ability to provide evidence for causality. They are also not ideal for testing theoretical arguments which imply long-term integration processes. The panels which include immigrants, while addressing the issue of longitudinal information, suffer from their own problems. In particular, panel *attrition* is considerably higher among immigrants compared to natives. It also varies dramatically across countries, which is relevant if cross-country comparisons are intended: for example, at least one-third of the immigrants included in a United States panel left the panel within the first decades (Warrent & Peck, 1980), while more than half left a UK panel after six years (Dustmann & Weiss, 2007) and one-quarter left a Swedish panel after five years (Edin et al., 2000). Equally important to mention is the fact that immigrants who remain in the panel are positively selected. This selection bias has been documented for Sweden (Edin et al., 2000), West Germany (Bellemare, 2004) and Canada (Picot & Piraino, 2013). In contrast to these data sources, registers provide accurate longitudinal data (thus addressing the shortcomings of cross-sectional datasets) and are less affected by refusal-driven attrition.

Registers are, however, affected by attrition due to natural causes, such as death and return migration. While death is likely to be adequately recorded, return migration is problematic for register data (and implicitly for studies based on them). Labour contracts, individual preferences, increased purchasing power, acquired human capital, risk diversification and relative deprivation are seen as important factors causing return migration. Earlier studies for Sweden have shown that the rate of return can vary widely between the various immigrant groups. These studies also argue that the rate of return depends heavily on the motivation to migrate. Labour migrants have generally a much higher rate of return than refugees (Klinthäll, 2007; Lundh & Ohlsson, 1999). Studies based on register data have several statistical techniques to control for outmigration and possible biases connected to this and therefore increase their reliability.

Although it can be easily agreed upon that registers provide high-quality data for integration research, an improvement therefore over other sources, some qualifications are needed. Firstly, the personal identity number in the Danish and Swedish statistical system is vital for the production of linked data; however, the central importance of this number is also a weakness of the system. If a person has not received a personal identity number, she or he will not be included in any of the regular statistical databases and an individual can only get a personal identification number if (s)he intends to stay for longer than one year (in Sweden) or three to six months (in Denmark) and has the legal right to do so. As a consequence, reliable data on short-term immigrants are missing. This also means that groups of immigrants, such as refugees or immigrants who have received a permit on other protective grounds, will be included in the population register and regular statistics only a couple of years after they have arrived in Sweden or Denmark. Secondly, since the registers include only the legally resident population, illegal immigrants, the undocumented or immigrants whose legal status has not been clarified are likely to be absent from this source. If a researcher is interested in capturing these categories, other identification methods need to be used.

Thirdly, the registers tend to over-cover foreign-born persons. This is due to the fact that there are no incentives to report to the tax authorities or municipalities that they are leaving the country. The over-coverage has been estimated at ca. 25-50000 persons, ie. around 4–8% of the total foreign-born population in Sweden (Statistics Sweden, 2016) while, in Denmark, it is estimated at about 7500 persons (0.14% of the total population, or 0.97% of the total immigrant population) (Statistics Denmark, 2017). Over-coverage is corrected *post-hoc* when the various administrative bodies identify that persons on their registers have emigrated.^8^

Last but not least, immigrant register data may be subject to biases absent from natives’ register data, because some of the data are self-reported and cannot be verified/triangulated with other sources. A typical example for this situation is completed education prior to immigration, which can be biased in two ways: it may be inaccurately reported by the immigrants themselves, or may be reported in categories from the countries of origin, which need to be equivalised with the host country’s education categories (Nielsen, Yazici, Petersen, Blaakilde, & Krasnik, 2012). It is recommended that researchers check whether the authorities managing the registers have rules in place to verify the reported information (for a discussion of such procedures, see Mørkeberg, 2000).

In the following sub-sections, three main uses of registers will be elaborated upon: as sources of statistical data on the immigrant population, as sources of data for register-based research on integration and as sampling frames.

### The use of registers: sources of statistical data

Registers are the main sources of information for population statistics. Although in 2007, the European Union has drawn up guidelines for population statistics to be collected by Eurostat,^9^ these guidelines are not always adopted when statistics are reported for domestic audiences. Therefore, researchers must be cautious when combining country data from different national sources. This section will elaborate upon the different categories and definitions used by the statistical institutes of Denmark and Sweden for classifying their respective non-native populations.

Statistics Denmark’s use of the Civil Registration System for statistical purposes is prescribed by the Law on Statistics Denmark (Lov om Danmarks Statistik §6 (jf. lovbekendtgørelse nr. 599 af 22. juni 2000). Statistics Denmark started to separately report data on immigrants from 1991 but, based on register data, statistics on this group can be obtained from much earlier. The information about immigrants and their descendants is comparable from 1980 onwards.^10^ In Sweden, the main laws governing the work of Statistics Sweden are the Official Statistics Acts (2001:99) and the Ordinance (1988:137) with the Directive for Statistics Sweden. The coordination of the various concepts to regulate statistical information has been enacted in Sweden since 1996 (MIS, 1996:5) and, since 2001, Statistics Sweden has the responsibility for statistical information on migration and asylum-seekers (Statistics Sweden, 2012).

**Fig. 1:**
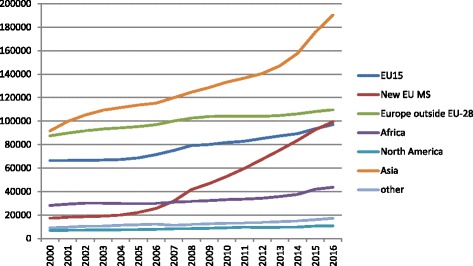
First-generation immigrant population in Denmark, 2000–2016, by broad area of origin. *Source*: Statistics Denmark

**Fig. 2:**
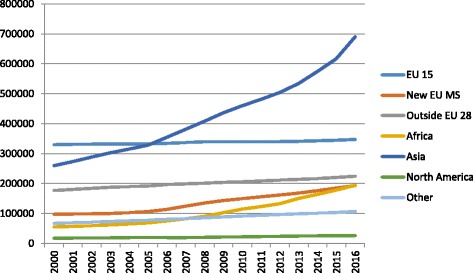
First-generation immigrant population in Sweden, 2000–2016, by broad area of origin. *Source*: Statistics Sweden

Statistics Denmark reported that 741,572 foreigners (570,581 first-generation immigrants and 170,991 descendants) were legally residing in Denmark at the end of 2016. The long-term trend shows a continuous increase over the last 15 years (see Fig. 1) for first generation immigrants, regardless of their area of origin. Two groups stand out: the increase of immigrants originating from Central and Eastern European countries from the mid-2000s onwards corresponds to these countries’ acquisition of EU membership. The increase in the number of immigrants from Asia reflects the recognition of refugees from war-torn countries. Statistics Sweden reported that 1,783,055 foreign-born individuals were legally residing in Sweden at the end of 2016 (see Fig. 2). The largest increases, from the mid-2000s on, are visible by the groups of immigrants originating in Asia (mainly from Iran, Iraq, Afghanistan and Syria) and Africa (mainly from Eritrea, Ethiopia and Somalia), driven by family reunifications and, more recently, by the recognition of refugees originating from these areas. Comparatively, the increase due to immigrants from the new EU member-states is relatively modest (though most additions to this group come from Poland and Romania).

Table 1 summarizes the main concepts used to categorize the non-native population in the official statistics of the two countries.

**Table 1:** Main terms and categories used to report on the non-native population in the official statistics of Denmark and Sweden

Main terms and definitions			
	Denmark	Sweden	Notes
Main terms	Immigrant Descendant Danish origin	Foreign-born Swedish background Foreign background	Categories not comparable; see below and Tables 2 and 3
Definition of main terms:	*• Immigrant*: ‘A person born abroad whose parents (or one of them if there is no available information on the other parent) are both foreign citizens or were born abroad. If there is no information available on either of the parents and the person was born abroad, the person is also defined as an immigrant’ (Statistics Denmark, 2017). *• Descendant*: ‘A person born in Denmark whose parents (or one of them if there is no information available on the other parent) are either immigrants or descendants with foreign citizenship. If there is no information available on either of the parents and the person in question is a foreign citizen, the person is also defined as a descendant’ (Statistics Denmark, 2017). *• Person of Danish origin*: ‘A person – regardless of place of birth – who has at least one parent who is a Danish citizen and was born in Denmark’ (Statistics Denmark, 2017).	No official definitions *•* Individuals born outside Sweden are reported as *foreign-born* (N1). *•* Children of immigrants born in Sweden are depicted as having a *foreign* or a *Swedish background* depending on the country of birth of the parents (N2). *•* Individuals with a Swedish background who were born in Sweden or foreign-born individuals with two or one Sweden-born parents. *•* Individuals with a foreign background who were either born in Sweden or are foreign-born and have two foreign-born parents. Foreign background is also used when Statistics Sweden publishes information on families and households.	In official statistics published by Statistics Sweden, the terms ‘immigrant’ and ‘descendant’ are not used; consequently no definitions similar to those used in Denmark are provided.
Categories on which statistical information is reported:			
Geographical area of origin	By country of origin (N3); continent; Western/non-Western (N4)	By country of origin; continent	
Citizenship	Citizenship	Citizenship	
Admission category	Admission category/residence permit (family reunification, work, education, *au pair*, interns, other, asylum)	admission category (refugee, family reunion, labour market, education, adoption, other)	
Time	–	*• Time in Sweden* is based on the date of national registration, with a reduction for time spent outside Sweden. *• Time since last immigration* is based on the last time an individual was registered in Sweden.	
Other	Age, gender and Danish residential area	Age, gender and Swedish residential area	
Additional information:			
Naturalization	Naturalization	Naturalization	
Categories not reported	Undocumented; illegal	Undocumented; illegal	
Asylum-seekers	Reported separately in the Aliens’ Register	Reported separately in the Asylum-seekers’ Register	Asylum-seekers count as immigrants when recognized as refugees (N5), which results in them receiving a residence permit, a personal identification number and registration in the regular population registers.

*Source*: The authors

N1: *Foreign-born* are divided by the duration of stay in two categories: individuals who have been in Sweden for less than five years or more than five years

N2: Children of foreigners are reported in three groups*:* born to two foreign-born parents, born to one parent born in Sweden and one foreign-born parent, and born to two Sweden-born parents

N3: Statistics Denmark (2017) uses the following criteria: ‘1) When no parents are known, the country of origin is defined from the person’s own information. If the person is an immigrant, it is assumed that the country of origin is equal to the country of birth. If the person is a descendant, it is assumed that the country of origin is equal to the country of citizenship. 2) When only one parent is known, the country of origin is defined as the country of birth of the parent. If this is Denmark, the country of citizenship is used. 3) When both parents are known, the country of origin is defined as the country of birth of the mother, respectively country of citizenship’

N4: *Western countries* include all the 28 EU countries, plus Andorra, Iceland, Liechtenstein, Monaco, Norway, San Marino, Switzerland, the Vatican State, Canada, the USA, Australia and New Zealand. *Non-Western countries* are all other countries (Statistics Denmark, 2017)

N5: A refugee is a person who, in fear for his/her life, finds him-or herself on the territory of another state than his/her own, and avails himself/herself of the protection of that state. An asylum-seeker is a refugee who officially lodges a claim for protection with the authorities of the state on whose territory he/she finds himself/herself. If a claim is accepted, the state extends one of several forms of protection (Under the 1951 Convention, humanitarian, temporary, etc), i.e the asylum-seeker receives a recognised refugee status

This summary description of the main concepts used by the Swedish and Danish authorities to collect and report data on immigrants (and emigrants) reveals the following:

- The two statistical authorities differ in their categorization of the population of non-Danish/non-Swedish ethnicity. While Denmark officially uses the terms ‘immigrants’ and ‘descendants’ and provides clear definitions for them, Sweden does not use these two terms. The Danish category ‘immigrants’ roughly corresponds to the ‘foreign-born’ category used in Sweden. However, the Swedish equivalent to Danish ‘descendants’ is more difficult to identify, as the Danish definition includes the citizenship of parents *and* their country of birth, while Swedish statistics divide children of immigrants across three different categories according to their parents’ place of birth. Out of the three categories, only that of children born in Sweden to foreign-born parents clearly overlaps with the corresponding part of the Danish ‘descendant’ category. Based on information on parents’ country of origin, researchers construct their own ‘descendant’ categories (see, for example, Andersson & Hammarstedt, 2010; Andersson, Obucina, & Scott, 2015; Hammarstedt & Palme, 2012). Tables 2 and 3 summarize the use of basic concepts by the statistical institutes of the two countries.
- Directly deriving from the previous point is the fact that the *data available on the web pages of the two statistical offices are not directly comparable*. Let us assume that someone is interested in seeing how many children with parents of immigrant background are in the two countries. On the Danish data page, by selecting ‘descendants’ one obtains the number of persons (children) born in Denmark from various types of family who have immigrant roots. On the Swedish data page, one has to select three categories (2 parents born abroad; 1 parent born abroad and 1 in Sweden; 2 parents born in Sweden). While the first two categories include children with immigrant roots, the third category is not as clear, because it includes all children of Swedish enthnicity *in addition to* children born to two parents of non-Swedish ethnicity, but who were born in Sweden (third generation).
- The terms *Danish origin* and *Swedish background* used by the two statistical authorities do not cover the same group: while, in Denmark, this category includes persons (regardless of place of birth) with at least one parent who is a Danish citizen *and* born in Denmark, the Swedish category is broader – as the parent does not need to have Swedish citizenship – but includes only persons born in Sweden. See Tables 2 and 3 below for a comparison.
- Statistics Denmark does not provide an official definition of the term ‘foreign background’. However, when the term is used in publications, it is constructed based on the country of origin of the person or his/her parents.
- Neither Danish nor Swedish official statistics include categories such as ‘minorities’, ‘ethnic groups’ or ‘religious groups’. This is different to countries such as the UK, where individuals are asked to assign themselves to certain ethnic groups. If the terms are used in publications, the country of origin is used as an identifier.
- Stock data based on the registers are not fully comparable across the two countries, as Denmark requires registration on the population register after three or six months, while in Sweden it is after one year (see below for details).
- A note on internationally reported data is needed. Data collected and presented by Statistics Denmark for *immigrants* are only partially comparable with Eurostat data on Denmark. The reason is Eurostat regulation 862/2007, which requires the statistical office to count/report a person as an immigrant only if she/he intends to stay for at least 12 months. Since Statistics Denmark uses register data, it counts/reports a person as an immigrant only if she/he intends to stay for more than three or six months, Eurostat’s numbers for immigration in Denmark are lower compared to those published by Statistics Denmark.^11^ This problem is absent in Sweden because the Swedish authorities include a newly arrived person on the population register only if (s)he intends to stay for more than one year. Thus, the *foreign-born* category reported by Statistics Sweden follows the Eurostat definition.

**Table 2:** Correspondence between concepts used by Statistics Denmark and the respective population groups

Place of birth of the individual	Parents		
	2 parents who are foreign citizens or 2 parents born abroad	2 parents who are Danish citizens born abroad	At least 1 parent who is a Danish citizen born in Denmark
Denmark	Descendant	Descendant	Danish origin
Abroad	Immigrant	Immigrant	Danish origin

*Source*: The authors

**Table 3:** Correspondence between concepts used by Statistics Sweden and the respective population groups

Place of birth of the individual	Parents		
	2 parents born abroad	1 parent born abroad and 1 born in Sweden	2 parents born in Sweden
Sweden	Foreign background	Swedish background	Swedish background
Abroad	Foreign-born/foreign background	Foreign background	Foreign background

*Source*: The authors

### The use of registers: data for immigration research

As most governmental and scholarly research in Denmark and Sweden is register-based (Sandberg, 2012), it comes as no surprise that a great deal of *immigration research* relies on register data (see Nørredam, Kastrup, & Helweg-Larsen, 2011 for an argument about the usefulness of register data for immigration research). Researchers can opt either to build their own datasets, taking advantage of the linkability of the registers, or to use the pre-set longitudinal databases which the Danish and Swedish statistical institutes offer for research. For example, the most important database for migration and integration research in Sweden is STATIV, while, in Denmark, IDA (Danish Integrated Database for Labour Market Research) includes background information to identify immigrants, and thereby enables the study of their labour-market incorporation (Timmermans, 2010). In the following, we survey English-language social-science immigration research,^12^ focusing on the type of research questions addressed in this literature and the use of register data.

#### A focus on integration outcomes

Studies in this vein present comparisons between natives and (various groups of) immigrants with regard to the outcomes of interest. Albeit not always explicitly formulated, they subscribe to an understanding of immigrants’ integration (some use the word assimilation) as the absence of/small differences between immigrants’ and natives’ attainment along the dimensions of interest. Studies in this category focus on outcomes such as labour-market participation and attainment, wages (Andersson & Hammarstedt, 2015; Bevelander, 2001, 2005; Blume, Ejrnæs, Nielsen, & Würtz, 2009; Edin et al., 2000; Helgertz, Bevelander, & Teganumataka, 2014; Husted et al., 2001; Ohlsson, Broomé, & Bevelander, 2012; Rosholm, Scott, & Husted, 2006), poverty (Blume, Gustafsson, Pedersen, & Verner, 2005, 2007; Blume & Verner, 2007), educational attainment (Andersson, Östh, & Malmberg, 2010; Dahlstedt, 2011; Dahlstedt & Bevelander, 2010; Nielsen, 2011), reliance of social safety net (Hammarstedt, 2000) or residential conditions and preferences (Bråmå, 2006; Edin, Fredriksson, & Åslund, 2003; Pendakur, Pendakur, & Bevelander, 2016; Piil Damm, 2009). The results are likely highly to be reliable both because they use very large samples (usually the entire immigrant population and a large sample of the native population) and because their detailed data on labour-market history, family background, socio-demographics and income allow the isolation of the effects of the variables of interest with precision.

A typical example for studies in this field is Rosholm et al. (2006), which uses panel register data from Sweden and Denmark over 10 years to explore the employment assimilation of immigrants from different countries of origin. The study finds that, although the labour market conditions in the two countries moved in different directions between 1985 and 1995, immigrants experienced a decline in employment prospects. This decline was experienced by immigrants from Norway, as well as from Poland, Iran, and Turkey, albeit at different rates. The authors conclude that more flexible employment forms, the move towards specialised skills and new forms of capital make immigrants less attractive on the labour market. Their findings confirm both Bevelander’s earlier research on the negative effects of structural changes on immigrants’ employment (Bevelander, 2001, 2005) and studies which find that immigrants arriving as refugees are particularly punished on the labour market (Blume et al., 2005; Husted et al., 2001). Other typical research questions of particular concern focus on immigrants’ educational attainment and its effect on their life chances. A recurrent result shows that immigrants have a higher risk of being over-educated (compared to natives) (Dahlstedt, 2011; Joona, Gupta, & Wadensjö, 2014), and that this risk may increase with the length of time spent in the host country and the number of unemployment spells (Nielsen, 2011).

#### A focus on integration as an inter-generational process

Studies which look at inter-generational integration processes focus on outcomes and/or on changes over time. First- and second-generation immigrants are compared, both to each other and to natives, across different ethnic groups. As the registers allow the identification of family members as well as their objective conditions (living arrangements, income and health status), the processes of interest can be traced at the family level and therefore the effects of family-related factors can be captured more accurately than it is usually the case through surveys. Most research is focused on earnings, eanings mobility and labour-market mobility (Andersson & Hammarstedt, 2010; Gustafsson, Katz, & Österberg, 2016; Hammarstedt & Palme, 2012; Österberg, 2000), educational attainment and trajectories (Behtoui, 2013; Bygren & Szulkin, 2010; Colding, 2006; Dahlstedt, 2015; Smith, Helgertz, & Scott, 2016), family formation and fertility decisions (Celikaksoy, 2012; Scott & Stanfors, 2011), and spatial mobility (Macpherson & Strömgren, 2013; Nielsen, 2016). For example, Nielsen (2016) draws on assimilation theories and aims to find evidence for both spatial and straight-line assimilation in the transition of leaving home in Denmark. The author compared Turks, Somalis and Danes and found intergenerational spatial mobility in all groups, which he interprets as evidence for straight-line assimilation. However, neighborhood characteristics affected mobility: the higher the share of non-ethnic Danes in the neighborhood, the lower the intergenerational mobility. This study illustrates well the power of register-based research to generate accurate analyses by providing ample data. To start with, the dataset spanned from 1986 to 2006, and included all the Turks and Somalis residing in Denmark and a random 7% sample of Danes. As to the data per se, the researcher had access to detailed information – such as income, educational level, social group, gender, type of family, number of family members, date of first leaving the parental home – on all individuals in the sample and, from the housing register, parental house conditions, such as tenure type and the dimensions of parental housing unit. Moreover, based on housing register and individual information, the percentage of ethnic minorities in the parental neighborhood could be calculated.

All in all, studies in these two categories share several characteristics, which give considerable weight to their reliability and validity: they rely on information on virtually the entire immigrant group of interest, a feature unparallelled by any public opinion survey. They bring in detailed register records which provide accurate information about the conditions and characteristics of individuals and their living and working contexts, and which allow the effects of the factors of interest to be accurately isolated. Longitudinal data allow researchers to observe long-term trends (sometimes over 20–30 years), which is also a feature difficult to attain with survey data which would be affected by recall bias.

Without denying the insights that these studies have brought forward, we have to recognize that register data uncover only a limited palette of possible integration processes, in particular those related to socio-economic integration. Cultural integration, preferences and attitudes, which are strong signifiers of attachment to the host country, cannot be researched through register data. Moreover, register-based studies are limited in their ability to explain the mechanisms behind some of the patterns observed. For example, Nielsen (2011) observed that age at migration increases the over-education risk for immigrants educated in Denmark, but lowers it for those educated abroad. The author speculated that this may be the effect of work experience that later migrants might have had before arriving in Denmark. Blume and Verner (2007) observed a strong effect of exiting from welfare dependency in the case of immigrants cohabiting with natives, and assumed this to be the result of immigrants using their partners’ networks to find employment and lift themselves off welfare dependency. In the conclusion of his study of spatial assimilation, Andersen (2010) hypothesized that some immigrants’ continued residence in multi-ethnic neighborhoods long after their socio-economic status improved is a signal of their attachment to the friends they made in the respective residential areas. However, in the absence of data about actual behaviors and preferences, none of these explanations could be tested and thus remained (highly credible) speculation. Arguably, in order to test such explanations, data must be collected via specifically designed studies which focus on immigrants’ opinions and preferences. In the next section, the use of registers to select immigrant samples for such studies is discussed. However, before this, we have to mention the third use of registers, namely as complements for survey data.

#### Complementing survey data

A good example of this use is the study by Jensen and Rasmunssen (2011) on the effect of immigrant concentration on the educational performance of immigrant and native children. The survey data were provided by compiling two Danish PISA studies which focused on children’s performance. The CPR numbers of the children interviewed in these PISA studies allowed the researcher to add register data regarding the children’s family background and contextual factors regarding their schools and their neighborhoods. Thus, they were able to add more variables to their study, which proved essential for constructing relevant instruments and reducing possible omitted variable bias in their models. Their results, which indicated a negative effect of immigrant concentration on the educational performance of children, were echoed in another study which used a different methodology and administrative panel data (Andersen & Thomsen, 2011). These findings indicate that combining register with survey data can be a successful strategy to increase the accuracy of the findings even when the survey provides only cross-sectional information. A similar strategy has been used by other studies: Nielsen et al.’s (2012) analysis of cross-border health care use by ethnic Danes and first- and second-generation Turks, found that respondents of Turkish origin were more likely than Danes to seek health care outside Denmark. Plenty and Jonsson (2017) combined the Swedish CILS4EU survey of adolescents and register data on family income and parents and found that students with immigrant backgrounds felt rejected more than majority youth and that first-generation non-European immigrants felt more isolated. Hjalmarsson (2017) uses a similar data combination strategy and finds that adolescents who recently arrived in Sweden are more likely to experience peer rejection than their Swedish counterparts.

Compared to the two previously mentioned uses of register data, this approach is used in fewer studies and not to its full potential. In spite of the availability of longitudinal register data, this aproach is not used to provide a longitudinal perspective on immigrants’ integration, either because, thus far, most immigrant survey data currently available are cross-sectional or because authors are using only one wave of panel studies (see, for example, Hjalmarsson, 2017; Plenty & Jonsson, 2017).

In spite of these limitations in current research combining register and survey data, we would like to encourage researchers and governments to see this combination as the way forward in immigration and integration research. We argue that, compared to the studies mentioned under the previous two categories, research following this approach has some undeniable advantages. For example, it brings to the forefront immigrants’ subjective experiences, thereby substantiating our understanding of integration processes. Experiences of social isolation and rejection (Hjalmarsson, 2017; Plenty & Jonsson, 2017) are not recorded in registers but they are of major importance in shaping the relationship between immigrants and host-countries societies, ultimately influencing the direction of their integration (see Berry, 1997). Moreover, one has to acknowledge that, albeit accurate, register data may be misleading, because they reflect only the interactions that individuals have with the institutions of the state which is maintaining the registers. It means that they do not capture a whole other range of behaviors, which may be equally relevant for assessing integration, and here surveys can make a difference. For example, Nielsen et al. (2012) show that immigrants are resorting to cross-border doctor visits more than native Danes, and uncover this by asking indiviudals about their health-care-related behavior. If only register data had been used for this study, the authors would have concluded that immigrants are healthier than native Danes, because the health register records fewer doctor visits by the former than by the latter. Unfortunately, the authors’ questionnaire did not allow them to explore the possible reasons for immigrants seeking health-care abroad, but they speculate that the reasons can range from a lack of knowledge about the Danish system to a good knowledge of the two systems – which allows people to pick and choose – and to a lack of trust in the Danish health-care system and personnel.

An essential step in developing research designs which combine register data with survey data is to develop ways of producing a reliable and high-quality sample of the population of interest, in this case immigrants. In the following section we turn our attention to this issue and discuss the use of population registers as sampling frames in Denmark and Sweden, emphasizing the strengths and the caveats of this approach.

### The use of registers: sampling immigrants

Theoretically, Danish and Swedish population registers are ideal sampling frames: they are directly accessible to researchers, they are centralized and they include a handful of key characteristics which allow the identification of the population of interest: age, gender, current address, country of origin, year of arrival. Thus, researchers can extract samples of immigrants which confidently fulfill the condition of ‘randomness’. Such samples can be extracted at the national, the local and the regional level. Moreover, population registers can subsequently be used both to weigh the data in case the population actually surveyed has been biased, and to survey the same sub-sample again (if the researcher is interested in creating a panel dataset). It goes without saying that using population registers as sampling frames is advantageous also for cost reasons: on the one hand, a centralized register implies one point of contact for the researcher; on the other, access to register data frees the researcher from the need to include socio-economic issues in the questionnaires, which can thus be shorter and focused on the issues of interest. Shorter questionnaires also limit bias due to an overload of respondents and can result in higher-quality answers.

However, there is a set of caveats directly related to the use of registers as sampling frames, as the authors’ discussions with survey experts in Denmark and Sweden have uncovered.^13^ Some of these may limit researchers’ ability to obtain samples of the desired quality, while others predefine the population in certain ways which may limit researchers’ ability to address some research questions. We start by mentioning the issue of *contact*. Although registers provide information about the addresses of potential respondents, not all of the latter may be reachable, due mainly to two situations:

- Permanent out-migration – as previously noted, registers probably include more immigrants than actually reside in the country because, in spite of the requirement to do so, many people emigrate from Denmark and Sweden without informing the relevant authorities.
- Temporary out-migration – there is no obligation to inform the register authorities either in Denmarkor in Sweden for departures shorter than six months. Compared to non-EU immigrants, those who are European are more likely to travel across borders or to temporarily return to their countries of origin. Thus, there is a risk that the person included in the sample cannot be contacted, a risk which is higher for EU than for non-EU immigrants. According to the experts interviewed, the risk is particularly high in the summer and around the main religious holidays, and lower in the rest of the year.

The discrepancy between the out-movement of immigrants and the register information at a given point in time^14^ can be problematic for obtaining a representative sample of immigrants. The problems are likely to increase if out-migration is not random. These contact problems can be further compounded by the method used in interviews. In particular, the risk is high if subjects are to be contacted by telephone, as the addresses sampled from the population register must be matched with telephone numbers – which are not fully available.

Secondly, using registers as sampling frames is an appropriate strategy only if the planned research targets *legal immigrants*. The population registers include only individuals who enter legally and intend to reside long-term in one of the two countries. For researchers who are interested in the processes and experiences of specific categories of immigrants – such as temporary immigrants, posted workers, illegal immigrants or refugees – other identification and sampling techniques are needed (see, for example, the use of respondent-driven sampling in Arnholtz & Wesley Hansen, 2013).

Thirdly, the use of registers as sampling frames may raise *data protection* and *privacy issues*. Researchers might appreciate the richness and linkability of register data, and their access to these sources may be granted on a firm legal basis. However, the individuals included in the sample might not feel the same way. Unlike native Danes and Swedes, who are accustomed to the register system and its availability to various authorities and researchers, immigrants may come from countries where registers either do not exist or are not available to non-state authorities, or where interactions with authorities are regarded with suspicion. Therefore, immigrants may become distrustful if information which they provide to state authorities can be accessed by researchers and used to single them out and contact them. This can negatively influence their willingness to take part in the survey, which ultimately may affect the representativeness of the sample.

Meta-information from existing immigrant surveys conducted in the two countries (Groenewold & Lessard-Phillips, 2012; UIM, 2013, 2014, 2016, 2017), as well as analyses of non-responses (Deding, Fridberg, & Jakobsen, 2008), illustrate that, even if immigrant surveys start with samples extracted from population registers, the final samples still suffer from pronounced non-reponse rates, which are much higher among immigrants than natives. Moreover, the various immigrant groups have different response rates: for example, the UIM surveys consistently report that immigrants over 30 years old have higher response rates compared to immigrants between aged between 18 and 29 years old, but both are significantly lower than the response rates of natives (between 40 and 50%, compared to ca. 60%) (UIM, 2013, 2014, 2016, 2017). Deding et al. (2008) found that language problems do not drive the high non-response rate among immigrants, but that immigrant groups behave differently in survey situations: Pakistanis have the highest non-contact rate compared to Turks and Iranians (due to their having moved and their failure to meet the interviewers), while Turks have the highest refusals rate (driven by refusals by the spouse-husband). Albeit not at high rates, refusals due to suspicion occur more among immigrants than among natives (Deding et al., 2008). The TIES study reports similar contact and refusal problems for second-generation Turks in Sweden, which prompted the research team to draw a second sample from the register (Groenewold & Lessard-Phillips, 2012).

## Discussion and conclusion

Researching the integration of immigrants is becoming increasingly relevant in all European countries. To produce high-quality integration research, scholars should have *access to data* about immigrants and the data should be *systematic* (collected on *large*/*representative* groups of immigrants and over time) and both *objective and subjective*.

The question asked at the beginning of this article was whether using the registers as data sources for integration research helps to improve the quality of information about immigrants and produces data that are close to these desiderata. In order to answer this question, the authors surveyed the different uses of register data in Denmark and Sweden – the most likely countries in which to study these due to their well-developed system of population registers.

Danish and Swedish population registers collect *systematic* data on the entire legally resident immigrant population. Researchers have direct *access* to the population registers and, through the statistical institutes of the two countries, can access linked data across different specialized registers. The latter provide detailed longitudinal information on individuals’ health status, labour-market trajectories, educational attainment or residence, allowing the reasearchers to implement sophisticated modelling techniques which track immigrants’ integration paths and identify their determinants. Such studies usually rely on data from the entire immigrant population of interest, and mainly produce comparisons between natives and the different groups of immigrants, which subscribes to a view of integration as the reduction of differences between the former and the latter. Without denying the valuable insights produced by such register-based studies, unseen aspects of the integration process, such as the adoption of values and reactions to the moral codes of the host societies, remain unexplored because they cannot be dealt with through the *objective* information that registers record. It becomes increasingly indisputable that a deeper understanding of the integration trajectories of immigrants needs high-quality *subjective* data as well. Although registers do not collect such data, they can be used as sampling frames for obtaining random representative immigrant samples.

Our discussion of registers as sampling frames has shown that, theoretically, they have the potential to produce *probabilistic samples* of immigrants, but there are several features which may affect the final sample quality. Centralized population registers provide researchers with a single access point to the sampling frame, which contains data on the entire legally resident population. Researchers can extract not only national but also sub-national random samples, as well as those of specific immigrant groups. However, the quality of a sample does not depend only on its randomness; it also depends on researchers’ ability to contact and successfully interview the randomly selected individuals. Our review has identified several features of registers which affect this ability. First, as there are few incentives to report out-migration, some addresses randomly selected might not correspond to the real location of individuals. It is likely that this invalid address problem is more pronounced for cetain groups, thereby biasing the sample (Deding et al., 2008). Second, immigrants may become suspicious when information which they provide to state authorities (ie. the registers) becomes available to third parties, and this may result in their refusal to take part in the survey. Depending on personal experiences, certain categories of immigrant are more likely to be suspicious, thereby introducing a bias in the sample. A third point should be also raised, although it does not reflect a weakness per se but, rather, brings to the fore a limitation with respect to the population of interest: registers do not contain the entire immigrant population but only those who have acquired legal residence status. Whether this is problematic or not depends greatly on the researcher’s intentions and conceptualizations. For example, if we argue that one can talk about integration only in the case of long-term immigrants, then sampling immigrants on the basis of population registers is satisfactory. However, if a person subscribes to the view that integration starts the moment an immigrant arrives in a foreign country, then sampling from a source which includes individuals some time after their arrival might not be enough.

In addition to their use for sampling, registers can have a positive impact on the overall data collected. Theoretically, the availability of a comprehensive dataset with objective information about each of the individuals included in the sample frees the researchers from the need to develop long questionnaires (thereby reducing the costs – always a bonus) and allows them to focus on attitudinal questions. Additionally, this strategy is likely to increase the quality of the data obtained through surveys, because respondents do not have to provide answers that might be subject to recall bias or which they are unwilling to report on.^15^

To sum up, therefore, Danish and Swedish population registers fulfill several of the conditions initially set out for good data for integration research: they provide *systematic*, long-term *objective* data about the entire legally resident immigrant population, which is fully *available* to researchers (under certain conditions). Using registers as sampling frames provides researchers with the opportunity to collect *subjective* data. However, the quality of these data for making inferences about the immigrant population largely depends on whether the sample extracted from the register is representative/probabilistic. As discussed above, there are several situations which are likely to *affect the representativeness* of the samples. Being aware of the potential limitations of registers as sampling frames means that researchers are able to take measures to limit possible bias. Therefore, we encourage more research reporting on non-responses or contact problems in immigrant surveys, as this would be helpful for devising tailored solutions.

We cannot conclude without commenting on the comparative potential of register-based research. Denmark and Sweden are similar in that they both make use of population registers to keep track of their legally resident population, that there are numerous registers which record information on the various aspects of life (such as health, employment, welfare support, health), that a person can be tracked through different registers via their personal identification number, and that researchers have access (under the conditions set by the data protection legislation) to these data, which are well documented and are largely comparable over time in each of the two countries. Given that country of birth and the family connection are always recorded, the researchers can identify members of the first, second and even third generations. Moreover, since the registers are centralized nationally and in-country residence information is updated rapidly, a variety of samples – of national, regional, particular ethnic group, only descendants or the entire immigrant population – can be extracted at relatively low cost.

These are solid common grounds which encourage cross-country comparative studies. However, this statement needs qualification: First, the comparative potential of register-based research is limited in scope. As the other articles in this issue show, the full availability of registers to researchers is limited in other European countries (ranging from its absence in Italy to very decentralized and/or difficult access in Germany) (see also Salentin & Schmeets, 2017; Sanguilinda et al., 2017). Thus, Denmark and Sweden (and the other Nordic countries) emerge as the primary group of countries where cross-country comparative research based on register data and immigrant register-based samples can be fully implemented at national as well as at sub-national levels. Depending on the research interest, the effect of various contextual factors on immigrants’ integration can be explored, as the Nordic countries share some features (for example, universal welfare regimes), while differing visibly in others (for example, immigration and integration regimes). Second, given the legal framework circumscribing access to a country’s register, cross-country comparative studies invite cross-border cooperation between research teams.

To conclude, this study has presented and discussed evidence in support of the idea that population registers in Denmark and Sweden are ideal as a resource for studying the integration of immigrants and their children as a process because the data which they offer are of a high quality, systematic, longitudinal and generational. They also offer the possibility to combine registers and to combine administrative data with survey data, opening the door for studies which focus on attitudinal questions and issues of further embeddedness. If researchers are aware of the caveats regarding the registers, they can use them to obtain representative samples of immigrants and their children, and develop research projects building on survey panels which, complementing the already available register information, would open up a new research track for testing complex integration theories.

## Footnotes

1 In this study we use the term ‘objective data’ (as opposed to ‘subjective data’) to refer to the characteristics of an individual’s life: employment status, income, date and place of birth, education and so on. The corresponding term used in the demographic literature is ‘descriptive’. Subjective data refer to an individual’s opinions, preferences and attitudes.

2 These are illustrative but very incomplete lists.

3 See https://www.understandingsociety.ac.uk/ (accessed 3 March, 2018).

4 Different categories of immigrants require different residence documents. EEA citizens can reside in Denmark for up to three months without a residence certificate and six months if the person is seeking employment. For longer stays, all EEA citizens have to request a *residence certificate* and must follow the rules included in the 2004 EU directive on freedom of movement and residence. All other foreigners need a *residence permit* in order to work or to stay more than three months in Denmark.

5 The formalities are spelled out (mostly in Danish) on the website of the Data Protection Agency (Datatilsynet, 2016). Researchers have direct access only to the main population register of the CRS. The linkage between registers is done only by Denmark Statistics, and researchers have access to an anonymized dataset.

6 Researchers are also advised to note that certain research-related services provided by Statistics Denmark may incur a fee (Statistics Denmark, 2016).

7 For example, the LINDA dataset may be available to EU researchers under certain conditions, determined by Statistics Sweden. See LINDA (2016). Researchers are advised to inquire about the conditions and possible costs with the organizations managing the data which interest them.

8 ‘Emigrants who do not plan to return to Denmark, do not have a strong incentive to report their emigration to the National Register of Persons. This will not be registered until a public authority attempts to make contact with the individual. In these instances the National Register of Persons must launch a thorough investigation in order to determine the new residence of the individual. If this residence cannot be determined, the individual is reported as a missing person instead of as emigrated. In other cases the emigration is registered when the individual re-immigrates’ (Statistics Denmark, 2017 http://www.dst.dk/en/Statistik/dokumentation/documentationofstatistics/immigrants-and-descendants (accessed 3 March, 2018).

9 For example, Eurostat collects data from the EU member-states which report migration statistics according to the guidelines set out in Regulation (EC) No. 862/2007. Eurostat data are thus comparable across EU countries from 2008 on, but not before, when reporting followed country definitions.

10 As of 1 July 2007, there has been a change in the processing of data that has made it possible to include information on all people once registered in the Central Population Register in the compilation of immigrants and descendants. This change made it possible to identify more parents and, in 2000, ‘descendants with origin in a western country’ was changed to ‘people of Danish origin’ (Statistics Denmark, 2017).

11 Statistics Denmark notes that data reported domestically follow the domestic definitions while, when data are reported to the EU, they are recalculated to reflect the definitions applied by Eurostat (see http://www.dst.dk/en/Statistik/dokumentation/documentationofstatistics/migration-to-and-from-denmark/comparability (accessed 3 March, 2018).

12 We conduct a non-exhaustive literature review of quantitative studies relying on register information using the entire population or large representative samples from Sweden and Denmark. We focus primarily on studies published in English in social-science journals, but we also include contributions published in collective volumes. We identify studies which contain keywords such as ‘immigrants’, ‘integration’, ‘assimilation’, ‘register data’ or ‘survey’. We include studies published in Danish or Swedish if they fulfill the search criteria. We recognize that many studies about immigrants in Denmark and Sweden have been published in other fields, for example, medicine (and we have included a couple of illustrations in our review), but our focus is on social-science contributions. Moreover, we recognize that there is an increased interest in surveying immigrants in the two countries of study, and several projects are in the making (see, for example, ‘When do children of immigrants strive?’, a collaboration between research centers at Åarhus and Malmö universities). However, our review includes only *finished/published* studies. We focus on studies published since 1990, as this time frame corresponds with an increased concern over immigrants’ integration in both Denmark and Sweden.

13 We are particularly grateful to Vibeke Jakobsen (SFI), Morten Sodeman (SDU), Mette Stensbek Christensen (epinion), Peter Linde, Jens Bjerre and Dorthe Larsen (DST) and Jens Arnholtz (FAOS) for their comments and information.

14 Discrepancies are (partially) corrected *post-factum* when emigrants inform the authorities or when the authorities discover the cases of out-migration.

15 Although we emphasize here the possibility to combine register and survey data, on a side note, we would like to add that register-based studies can be combined with qualitative interviews of immigrants. This strategy would allow researchers to produce reliable and valid results generalizable to the entire immigrant population, while in-depth interviews would give immigrants a voice and bring their life worlds into the limelight.

## References

[CR1] Aleksynska M (2011). Civic participation of immigrants in Europe: Assimilation, origin, and destination country effects. European Journal of Political Economy.

[CR2] Algan Y, Dustmann C, Glitz A, Manning A (2010). The economic situation of first and second-generation immigrants in France, Germany and the United Kingdom. The Economic Journal.

[CR3] Andersen HS (2010). Spatial assimilation in Denmark? Why do immigrants move to and from multi-ethnic neighbourhoods?. Housing Studies.

[CR4] Andersen SC, Thomsen MK (2011). Policy implications of limiting immigrant concentration in Danish public schools. Scandinavian Political Studies.

[CR5] Andersson E, Östh J, Malmberg B (2010). Ethnic segregation and performance inequality in the Swedish school system: A regional perspective. Environment and Planning A.

[CR6] Andersson G, Obucina O, Scott K (2015). Marriage and divorce of immigrants and descendants of immigrants in Sweden. Demographic Research.

[CR7] Andersson L, Hammarstedt M (2010). Intergenerational transmissions in immigrant self-employment: Evidence from three generations. Small Business Economics.

[CR8] Andersson L, Hammarstedt M (2015). Ethnic enclaves, networks and self-employment among Middle Eastern immigrants in Sweden. International Migration.

[CR9] Arnholtz J, Wesley Hansen N (2013). Labour market specific institutions and the working conditions of labour migrants: The case of Polish migrant labour in the Danish labour market. Economic and Industrial Democracy.

[CR10] Baadsgaard M, Quitzau J (2011). Danish registers on personal income and transfer payments. Scandinavian Journal of Public Health.

[CR11] Behtoui A (2013). Incorporation of children of immigrants: The case of descendants of immigrants from Turkey in Sweden. Ethnic and Racial Studies.

[CR12] Bellemare, C. (2004). *Identification and estimation of economic models of outmigration using panel attrition* (IZA DP No. 1065). Bonn: IZA. Retrieved from http://citeseerx.ist.psu.edu/viewdoc/download?doi=10.1.1.422.268&rep=rep1&type=pdf.

[CR13] Berk R (1983). An introduction to sample selection bias in sociological data. American Sociological Review.

[CR14] Berry J (1997). Immigration, acculturation, and adaptation. Applied Psychology: An International Review.

[CR15] Bevelander P (2001). Getting a foothold: Male immigrant employment integration and structural change in Sweden, 1970–1995. Journal of International Migration and Integration.

[CR16] Bevelander P (2005). The employment status of immigrant women: The case of Sweden. International Migration Review.

[CR17] Blume K, Ejrnæs M, Nielsen HS, Würtz A (2009). Labor market transitions of immigrants with emphasis on marginalization and self-employment. Journal of Population Economics.

[CR18] Blume K, Gustafsson B, Pedersen PJ, Verner M, Borjas G, Cripps J (2005). A tale of two countries: Poverty among immigrants in Denmark and Sweden since 1984. Poverty, international migration and asylum.

[CR19] Blume K, Gustafsson G, Pedersen P, Verner M (2007). At the lower end of the table: Determinants of poverty among immigrants to Denmark and Sweden. Journal of Ethnic and Migration Studies.

[CR20] Blume, K., & Verner, M. (2007). Welfare dependency among Danish immigrants. *European Journal of Political Economy*, *23*(2), 453–471.

[CR21] Bøcker Pedersen CB, Gøtzsche H, Møller JØ, Mortensen PB (2006). The Danish civil registration system: A cohort of eight million persons. Danish Medical Bulletin.

[CR22] Bråmå Å (2006). ‘White flight’? The production and reproduction of immigrant concentration areas in Swedish cities, 1990–2000. Urban Studies.

[CR23] Bygren M, Szulkin R (2010). Ehnic environment during childhood and the educational attainment of immigrant children in Sweden. Social Forces.

[CR24] Celikaksoy A (2012). Intergenerational transmission of interethnic union formation patterns in Sweden. Migration Letters.

[CR25] Colding B (2006). A dynamic analysis of educational progression of children of immigrants. Labour Economics.

[CR26] Crul M (2013). Snakes and ladders in educational systems: Access to higher education for second-generation Turks in Europe. Journal of Ethnic and Migration Studies.

[CR27] Crul M, Doomernik J (2003). The Turkish and Moroccan second generation in the Netherlands: Divergent trends between and polarization within two groups. International Migration Review.

[CR28] Crul M, Schneider J (2010). Comparative integration context theory: Participation and belonging in new diverse European cities. Ethnic and Racial Studies.

[CR29] Crul, M., Schneider, J., & Lelie, F. (2012). *The European second generation compared. Does the integration context matter?* Amsterdam: University Press.

[CR30] Crul M, Vermeulen H (2003). The future of the second generation: The integration of migrant youth in six European countries. International Migration Review.

[CR31] Dahlstedt I (2011). Occupational match: Over- and undereducation among immigrants in the Swedish labour market. Journal of International Migration and Integration.

[CR32] Dahlstedt I (2015). Over-education amongst the children of immigrants in Sweden. Nordic Journal of Migration Research.

[CR33] Dahlstedt I, Bevelander P (2010). General versus vocational education and employment integration of immigrants in Sweden. Journal of Immigrant and Refugee Studies.

[CR34] Datatilsynet. (2016). *Hvornår skal forskningsprojekter anmeldes til Datatilsynet* [*When should research project request permissions and be registered with the Data protection Agency*]. Retrieved from https://www.datatilsynet.dk/offentlig/forskning/hvornaar-skal-forskningsprojekter-anmeldes-til-datatilsynet/. Accessed 2 Mar 2018.

[CR35] de Rooij EA (2012). Patterns of immigrant political participation: Explaining differences in types of political participation between immigrants and the majority population in Western Europe. European Sociological Review.

[CR36] De Vroome T, Coenders M, van Tubergen F, Verkuyten M (2011). Economic participation and national self-identification of refugees in the Netherlands. International Migration Review.

[CR37] De Vroome T, Martinovic B, Verkuyten M (2014). The integration paradox: Level of education and immigrants’ attitudes towards natives and the host society. Cultural Diversity and Ethnic Minority Psychology.

[CR38] Deding M, Fridberg T, Jakobsen V (2008). Non-response in a survey among immigrants in Denmark. Survey Research Methods.

[CR39] Dinesen PT, Hooghe M (2010). When in Rome, do as the Romans do: the acculturation of generalized trust among immigrants in Western Europe. International Migration Review.

[CR40] Dribe M, Lundh C (2008). Intermarriage and immigrant integration in Sweden. Acta Sociologica.

[CR41] Dustmann C, Weiss Y (2007). Return migration: Theory and empirical evidence. British Journal of Industrial Relations.

[CR42] Edin PA, Fredriksson P, Åslund O (2003). Ethnic enclaves and the economic success of immigrants: Evidence from a natural experiment. Quarterly Journal of Economics.

[CR43] Edin PA, LaLonde RJ, Åslund O (2000). Emigration of immigrants and measures of immigrant assimilation: evidence from Sweden.

[CR44] Ersanili E, Koopmans R (2011). Do immigrants’ integration policies matter? A three-country comparison among Turkish immigrants. West European Politics.

[CR45] Eurostat (1995). Statistics on persons in Denmark. A register-based statistical system.

[CR46] Fassmann H, Reeger U, Sievers W (2009). Statistics and reality: Concepts and measurements of migration in Europe.

[CR47] Fleischmann F, Phalet K, Klein O (2011). Religious identification and politicization in the face of discrimination: Support for political Islam and political action among the Turkish and Moroccan second generation in Europe. British Journal of Social Psychology.

[CR48] Fokkema T, de Haas H (2015). Pre- and post-migration determinants of socio-cultural integration of African immigrants in Italy and Spain. International Migration.

[CR49] Font J, Mendez M (2013). Surveying ethnic minorities and immigrant population.

[CR50] Groenewold G, Lessard-Phillips L, Crul M, Schneider J, Lelie F (2012). Research methodology. The European second generation compared. Does the integration context matter?.

[CR51] Gustafsson, B., Katz, K., & Österberg, T. (2016). *Residential segregation from generation to generation: Intergenerational association in social spatial context among visible minorities and the majority population in metropolitan Sweden*. (IZA DP No. 9837). Bonn: Germany. Retrieved from http://ftp.iza.org/dp9837.pdf.

[CR52] Güveli A (2015). Are movers more religious than stayers? Religiosity of European majority, Turks in Europe and Turkey. Review of Religious Research.

[CR53] Hammarstedt M (2000). The receipt of transfer payments by immigrants in Sweden. International Migration.

[CR54] Hammarstedt, M., & Palme, M. (2012). Human capital transmission and the earnings of second-generation immigrants in Sweden. *Journal of Development and Migration 1*. 10.1186/2193-9039-1-4.

[CR55] Helgertz J, Bevelander P, Teganumataka A (2014). Naturalization and earnings: A Denmark–Sweden comparison. European Journal of Population.

[CR56] Hjalmarsson S (2017). Poor kids? Economic resources and adverse peer relations in a nationally representative sample of Swedish adolescents. Journal of Youth and Adolescence.

[CR57] Husted L, Nielsen HS, Rosholm M, Smith N (2001). Employment and wage assimilation of male first-generation immigrants in Denmark. International Journal of Manpower.

[CR58] Jakobsen V, Larsen L (2010). Does the causal effect of health on employment differ for immigrants and natives?.

[CR59] Jensen P, Rasmunssen AW (2011). The effect of immigrant concentration in schools on native and immigrant children’s reading and maths skills. Economics of Education Review.

[CR60] Joona, P. A., Gupta, N. D., & Wadensjö, E. (2014). Overeducation among immigrants in Sweden: Incidence, wage effects and state dependence. *IZA Journal of Migration, 3*. Retrieved from https://izajodm.springeropen.com/articles/10.1186/2193-9039-3-9.

[CR61] Just A, Anderson CJ (2014). Dual allegiances? Immigrants’ attitudes toward immigration. The Journal of Politics.

[CR62] Klinthäll M (2007). Refugee return migration: Return migration from Sweden to Chile, Iran and Poland 1973–1996. Journal of Refugee Studies.

[CR63] Kogan I (2006). Labour markets and economic incorporation among recent immigrants in Europe. Social Forces.

[CR64] LINDA. (2016). *General information*. Retrieved from http://www.jpi-dataproject.eu/Home/Database/78?topicId=2. Accessed on 2 Mar 2018.

[CR65] Lundh, C., & Ohlsson, R. (1999). *Från arbetskraftsmigration till flyktinginvandring* [*From labor migration to refugee immigration*]. Stockholm: SNS förlag.

[CR66] Luttmer EFP, Singhal M (2011). Culture, context, and the taste for redistribution. American Economic Journal: Economic Policy.

[CR67] Macpherson RA, Strömgren M (2013). Spatial assimilation and native partnership: Evidence of Iranian and Iraqi immigrant mobility from segregated areas in Stockholm, Sweden. Population, Space and Place.

[CR68] MIS. (1996:5). Riktlinjer för hur personer med utländsk bakgrund redovisas i statistiken, Migration Agency, Sweden [Guidelines for reporting people with a foreign background in statistics]. Migration Agency, Sweden.

[CR69] Morales L, Giugni M (2011). Social capital, political participation and migration in Europe. Making multicultural democracy work?.

[CR70] Mørkeberg H (2000). Indvandrernes uddannelse (the educational attainment of immigrants—In Danish).

[CR71] Nielsen CP (2011). Immigrant over-education: Evidence from Denmark. Journal of Population Economics.

[CR72] Nielsen RS (2016). Straight-line assimilation in leaving home? A comparison of Turks, Somalis and Danes. Housing Studies.

[CR73] Nielsen SS, Yazici S, Petersen SG, Blaakilde AL, Krasnik A (2012). Use of cross-border healthcare services among ethnic Danes, Turkish immigrants and Turkish descendants in Denmark: A combined survey and registry study. BMC Health Services Research.

[CR74] Nørredam M, Kastrup M, Helweg-Larsen K (2011). Register-based studies on migration, ethnicity, and health. Scandinavian Journal of Public Health.

[CR75] OECD (2015). Indicators of immigrant integration 2015. Settling in.

[CR76] Ohlsson H, Broomé P, Bevelander P (2012). Self-employment of immigrants and natives in Sweden: A multilevel analysis. Entrepreneurship and Regional Development.

[CR77] Österberg T (2000). Intergenerational income mobility in Sweden: What do TAX data show?. Review of Income and Wealth.

[CR78] Pendakur K, Pendakur R, Bevelander P (2016). Are residential and workplace concentration correlated for immigrants? Evidence for Sweden. Journal of International Migration and Integration.

[CR79] Petersson F, Baadsgaard M, Thygesen LC (2011). Danish registers on personal labour market affiliation. Scandinavian Journal of Public Health.

[CR80] Phalet K, Schönpflug U (2001). Intergenerational transmission of collectivism and achievement values in two acculturation contexts. Journal of Cross-Cultural Psychology.

[CR81] Pichler F (2011). Success on European labor markets: A cross-national comparison of attainment between immigrant and majority populations. International Migration Review.

[CR82] Picot G, Piraino P (2013). Immigrant earnings growth: Selection bias or real progress?. Canadian Journal of Economics.

[CR83] Piil Damm A (2009). Determinants of recent immigrants’ location choices: Quasi-experimental evidence. Journal of Population Economics.

[CR84] Plenty S, Jonsson JO (2017). Social exclusion among peers: The role of immigrant status and classroom immigrant density. Journal of Youth and Adolescence.

[CR85] Reigneveld S, Stronks K (1999). The impact of response bias on estimates of health care utilization in a metropolitan area: The use of administrative data. International Journal of Epidemiology.

[CR86] Röder A, Mühlau P (2011). Discrimination, exclusion and immigrants’ confidence in public institutions in Europe. European Societies.

[CR87] Röder A, Mühlau P (2012). Low expectations or different evaluations: What explains immigrants’ high levels of trust in host-country institutions?. Journal of Ethnic and Migration Studies.

[CR88] Röder A, Mühlau P (2014). Are they acculturating? Europe’s immigrants and gender egalitarianism. Social Forces.

[CR89] Rosholm M, Scott K, Husted L (2006). “The times they are a-changin”: Declining immigrant employment opportunities in Scandinavia. International Migration Review.

[CR90] Sahl Andersen J, de Fine Olivarius N, Krasnik A (2011). The Danish national health service register. Scandinavian Journal of Public Health.

[CR91] Salentin, K., & Schmeets, H. (2017). Sampling immigrants in the Netherlands and Germany. *Comparative Migration Studies*, *5*. 10.1186/s40878-017-0062-2. 10.1186/s40878-017-0062-2 PMC5730621 29264233

[CR92] Sandberg, M. (2012). *Reinforced Nordic collaboration on data resources: Challenges from six perspectives*. Retrieved from https://norden.diva-portal.org/smash/get/diva2:702828/FULLTEXT01.pdf.

[CR93] Sanguilinda, I. S., Barbiano di Belgiojoso, E., Ferrer, A. G., Rimoldi, S. M. L., & Blangiardo, G. C. (2017). Surveying immigrants in Southern Europe: Spanish and Italian strategies in comparative perspective. *Comparative Migration Studies*, *5*. 10.1186/s40878-017-0060-4. 10.1186/s40878-017-0060-4 PMC5670187 29152455

[CR94] Schmidt M, Pedersen L, Sørensen HT (2014). The Danish civil registration system as a tool in epidemiology. European Journal of Epidemiology.

[CR95] Schmidt-Catran A, Careja R (2017). Institutions, culture and migrants’ preference for state-provided welfare: Longitudinal evidence from Germany. Journal of European Social Policy.

[CR96] Scott K, Stanfors M (2011). The transition to parenthood among the second generation: Evidence from Sweden, 1990–2005. Advances in Life Course Research.

[CR97] Silberman R, Alba R, Fournier I (2007). Segmented assimilation in France? Discrimination in the labour market against the second generation. Ethnic and Racial Studies.

[CR98] Smith, C. D., Helgertz, J., & Scott, K. (2016). Parents’ years in Sweden and children’s educational performance. *IZA Journal of Migration, 5*. Retrieved from https://izajodm.springeropen.com/articles/10.1186/s40176-016-0054-2.

[CR99] Statistics Denmark. (2016). *Data for research*. Retrieved from http://www.dst.dk/en/TilSalg/Forskningsservice. Accessed 2 March 2018.

[CR100] Statistics Denmark. (2017). *Documentation of statistics for immigrants and descendants 2017 month 01*. Retrieved from https://www.dst.dk/en/Statistik/dokumentation/documentationofstatistics/immigrants-anddescendants. Accessed 2 March 2018.

[CR101] Statistics Sweden (2012). Official Statistics of Sweden - Annual Report 2012.

[CR102] Statistics Sweden (2016). *Overcoverage in the Total Population Register – a register study*. Stockholm: Statistics Sweden, Population and Welfare Department. Retrieved from http://www.scb.se/Upload/NSM2016/theme1/Tor%20Bengtsson%20-%20Stina%20%C3%85sling%20R%C3%B6nning.pdf.

[CR103] Timmermans, B. (2010). *The Danish integrated database for labor market research: towards demystification for the English speaking audience* (DRUID Working Paper, pp. 10–16). Copenhagen: Danish Research Unit for Industrial Dynamics.

[CR104] UIM. (2013). *Medborgerskab, Ligebehandling og Selvbestemmelse i Danmark. Det nationale integrationsbarometer* [*Citizenship, Equal Treatment and Self-determination in Denmark. The National Integration Barometer*]. Retrieved from https://integrationsbarometer.dk/tal-og-analyser/filer-tal-oganalyser/medborgerskab-ligebehandling-og-selvbestemmelse-i-danmark-2015. Accessed 2 Mar 2018.

[CR105] UIM. (2014). *Medborgerskab, Ligebehandling og Selvbestemmelse i Danmark 2014* [*Citizenship, Equal Treatment and Self-determination in Denmark 2014*]. Retrieved from https://integrationsbarometer.dk/tal-og-analyser/filer-tal-og-analyser/medborgerskabsrapport-2014. Accessed 2 Mar 2018.

[CR106] UIM. (2016). *Medborgerskab, ligebehandling og selvbestemmelse i Danmark 2016* [*Citizenship, Equal Treatment and Self-determination in Denmark 2016*]. Retrieved from https://integrationsbarometer.dk/tal-og-analyser/filer-tal-og-analyser/medborgerskab-ligebehandling-og-selvbestemmelse-i-danmark-2016. Accessed 2 Mar 2018.

[CR107] UIM. (2017). *Medborgerskab 2017. Baggrundstabeller fra Medborgerskabsundersøgelsen 2017* [*Citizenship 2017. Background tables from the Citizenship Survey 2017*]. Retrieved from https://integrationsbarometer.dk/tal-og-analyser/filer-tal-og-analyser/medborgerskab-2017-baggrundstabeller. Accessed 2 Mar 2018.

[CR108] UN (2007). Register-based statistics in the Nordic countries. Review of best practices with focus on population and social statistics.

[CR109] Van der Zwan R, Bles P, Lubbers M (2017). Perceived migrant threat among migrants in Europe. European Sociological Review.

[CR110] Van Tubergen F, Maas I, Flap M (2004). The economic incorporation of immigrants in 18 Western societies: Origin, destination, and community effects. American Sociological Review.

[CR111] Verkuyten M, Martinovic B (2012). Immigrants’ national identification: Meanings, determinants, and consequences. Social Issues and Policy Review.

[CR112] Vermeulen H (2010). Segmented assimilation and cross-national comparative research on the integration of immigrants and their children. Ethnic and Racial Studies.

[CR113] Warrent, R., & Peck, J. M. (1980). Foreign-born emigration from the United States: 1960–1970. *Demography*, *17*(1), 71–84. 7353709

